# Introducing online multi-language video animations to support patients’ understanding of cardiac procedures in a high-volume tertiary centre

**DOI:** 10.1093/ehjdh/ztae073

**Published:** 2024-10-01

**Authors:** Jude Almutawa, Peter Calvert, David Wald, Vishal Luther

**Affiliations:** Department of Cardiology, Liverpool Heart and Chest Hospital, Thomas Dr, Liverpool, L14 3PE, UK; Liverpool Centre for Cardiovascular Science, William Henry Duncan Building, University of Liverpool, 6 West Derby Street, Liverpool, L69 7TX, UK; Department of Cardiology, Liverpool Heart and Chest Hospital, Thomas Dr, Liverpool, L14 3PE, UK; Liverpool Centre for Cardiovascular Science, William Henry Duncan Building, University of Liverpool, 6 West Derby Street, Liverpool, L69 7TX, UK; Population Health Research Institute St George’s, University of London, London, UK; Department of Cardiology, Liverpool Heart and Chest Hospital, Thomas Dr, Liverpool, L14 3PE, UK; Liverpool Centre for Cardiovascular Science, William Henry Duncan Building, University of Liverpool, 6 West Derby Street, Liverpool, L69 7TX, UK

## The problem

The treatment options available to patients in clinical cardiology are diverse, many of which include invasive procedures. Shared decision-making between clinician and patient involves providing patients with information around risks, benefits, and alternatives, along with adequate time to process this. Exploring these elements takes time, which may be limited in busy, publicly funded healthcare systems. It is well-established that patients remember only a fraction of the information given during medical encounters.^1^

## A solution

The value of video animation in aiding procedural understanding is well established. Previous reports have shown improved patient-reported complete understanding of procedures (from ∼30 to 80%), in both elective and urgent cardiac clinical settings.^2,3^ This approach is transferable across medical and surgical specialties, showing similar results in general and thoracic surgery.^2^ Earlier studies demonstrated how this has led to a 56% reduction in non-attendance for implantable loop recorders^4^ and a 70% reduction in complaints and serious incidents due to failure to inform before consent across 10 cardiac and cardiothoracic procedures.^5^ Given that the video animations can be repeatedly accessed on any device, they can be shared with relatives, allowing for family involvement.

## Explain My Procedure

‘Explain My Procedure’ (EMP) creates and hosts short, multi-language narrated video animations that explain procedures in cardiac intervention, electrophysiology, and structural and cardiac surgery in lay terms to support shared decision-making prior to consent. An example is provided at www.explainmyprocedure.com/heart. The content is accessible online on any device with an Internet connection and can be watched multiple times, allowing patients ample opportunity to review the information and show it to relatives.

## The Liverpool experience

**Figure 1:**
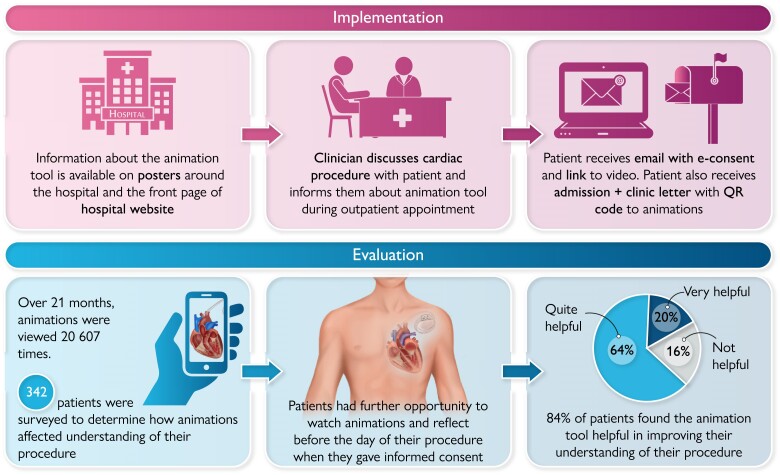
Implementation and patients’ evaluation of online narrated video animation tool. Images courtesy of Explain My Procedure©, reproduced with permission.

Liverpool Heart and Chest Hospital is a high-volume tertiary cardiothoracic centre, accepting patients across the north-west of England and Wales, with a 3 million adult patient catchment area including all socio-economic backgrounds. In April 2022, we purchased unlimited patient access to 31 video animations within the EMP platform in three languages (English, Welsh, and Polish). We systematically incorporated these into practice. Web links and QR codes for each video animation were provided and embedded within the patient pathway, using existing communication opportunities in advance of the patient’s procedure. This included in-person communication during the outpatient doctor-led cardiology clinic and during their nurse-led pre-assessment clinics, as well as via e-mail and/or post. We also signposted the resource using posters in the outpatient department and on our hospital website. *Figure 1* shows a typical patient pathway.

Between April 2022 and January 2024, there were ∼20 607 animation views, with 90% watched to completion. The three most viewed animations were ‘angiogram/angioplasty’ (4135 views), atrial fibrillation ablation (1415 views), and transcatheter aortic valve implantation (TAVI; 1243 views). We obtained feedback from a small consecutive sample (342 patients) attending our nurse-led pre-assessment clinic using a 3-point Likert scale (very helpful, quite helpful, or not helpful). Two hundred and eighty-eight patients (84%) felt the procedural video animations were very helpful or quite helpful. Of those who did not find the videos helpful (54 patients), anecdotally reported reasons included ‘not wanting to know’ or ‘feeling already adequately informed’.

Not all patients were aware of the availability of the resource in advance of the clinic. We have since furthered awareness of the resource among patients by incorporating links directly within outpatient clinic letters to upstream the information prior to pre-assessment, allowing patients longer to view, learn, and formulate questions that may matter to them, which can be posed at the pre-assessment clinic.

## Conclusion

Digital animation tools can be easily incorporated within a high-volume tertiary cardiac centre to support shared decision-making and increase equality, diversity, and inclusivity. More than four-fifths of all patients found video animations about their cardiac procedure helpful in increasing their understanding prior to consent.

## Data Availability

Anonymised data can be made available upon reasonable request.
